# The “polymorphous” history of a polymorphous skull bone: the sphenoid 

**DOI:** 10.1007/s12565-017-0399-5

**Published:** 2017-03-27

**Authors:** Claudia Costea, Serban Turliuc, Andrei Cucu, Gabriela Dumitrescu, Alexandru Carauleanu, Catalin Buzduga, Anca Sava, Irina Costache, Dana Turliuc

**Affiliations:** 1 Gr. T. Popa University of Medicine and Pharmacy, Iasi, Romania; 2 Nicolae Oblu Emergency Clinical Hospital, Iasi, Romania

**Keywords:** Anatomy, History, Sella turcica, Skull base, Sphenoid bone

## Abstract

For a long time, because of its location at the skull base level, the sphenoid bone was rather mysterious as it was too difficult for anatomists to reach and to elucidate its true configuration. The configuration of the sphenoid bone led to confusion regarding its sutures with the other skull bones, its shape, its detailed anatomy, and the vascular and nervous structures that cross it. This article takes the reader on a journey through time and space, charting the evolution of anatomists’ comprehension of sphenoid bone morphology from antiquity to its conception as a bone structure in the eighteenth century, and ranging from ancient Greece to modern Italy and France. The journey illustrates that many anatomists have attempted to name and to best describe the structural elements of this polymorphous bone.

## Introduction

Anatomy is “the oldest child of Mother Medicine” (Tubbs 2014), and there is no doubt that it has been the basis for the amazing progress made in the science of human healing. The efforts of anatomists to identify and describe any bone, muscular, vascular, or nervous structure should be recognized as major contributions to scientific research, and this efforts proved their scientific interest (Kataoka et al. 2007).

During the first period of development of the field of anatomy, physicians only described the anatomical structures that they could see. They named those structures by analogy, using the similarities between the shapes of the newly discovered structures and the shapes of different objects in their environment (Turliuc et al. 2016a), their architecture (Turliuc et al. 2017), or their civilization (Turliuc et al. 2016b), meaning that every anatomical term is a “historical construction” (Arráez-Aybar et al. 2015).

Located deep at the skull base level, the sphenoid bone is an anatomical structure that was, for a long time, difficult for anatomists to reach. The history of anatomical descriptions and the names of the entire sphenoid bone and its numerous components is quite complicated but also fascinating.

In this article, we take the reader on a journey across time and space, charting the development of anatomists’ comprehension of sphenoid bone morphology—from antiquity to its conception as a bone structure in the eighteenth century, and from ancient Greece to modern Italy and France.

## The anatomical conformation of the sphenoid bone

**Fig. 1:**
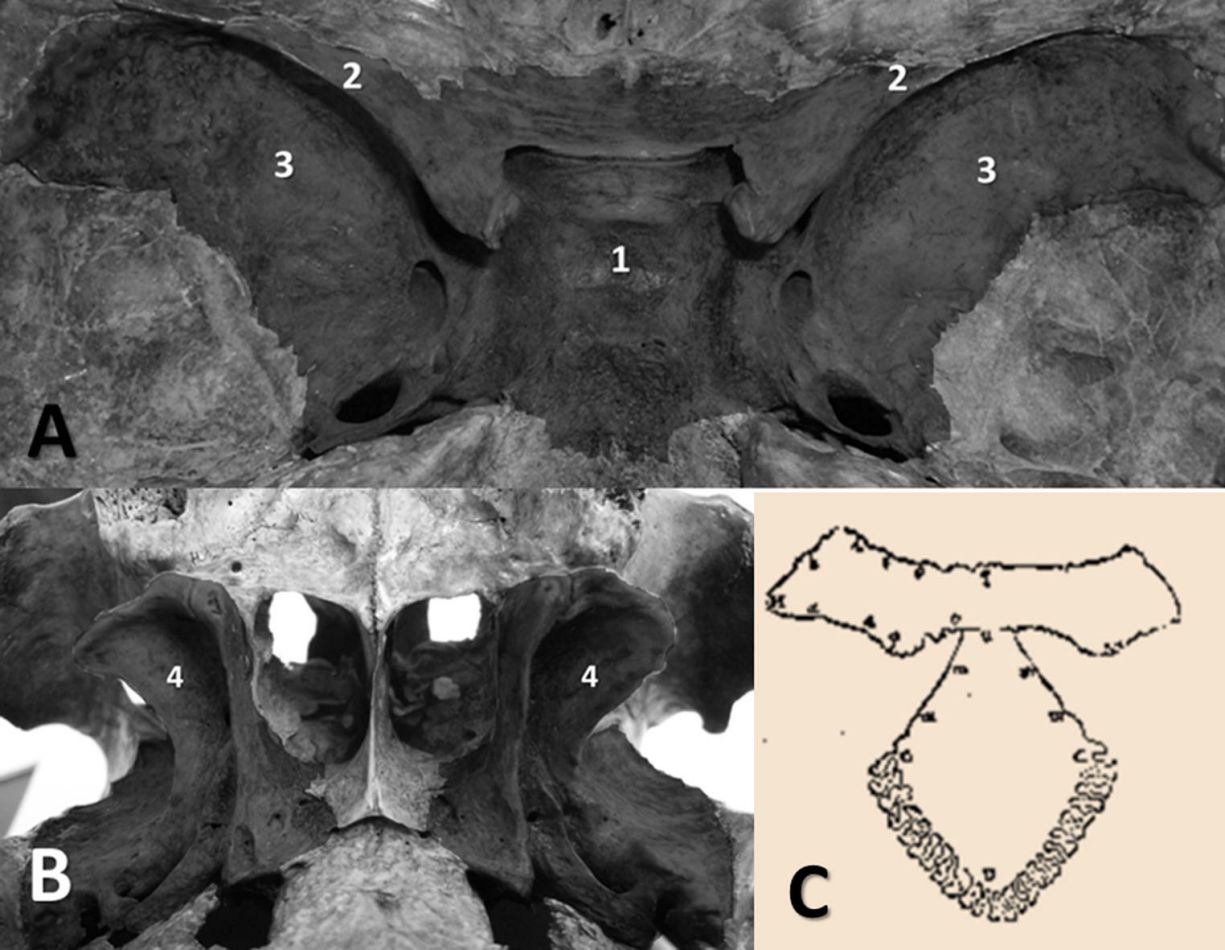
**a** Endocranial view of the sphenoid bone, showing the body (*1*), lesser wings (*2*), and greater wings (*3*). **b** Exocranial view of the sphenoid bone with the pterygoid processes (*4*) (Dr. A. Iordache’s personal collection). **c** Vesalius’s drawing of the sphenoid bone contour (adapted from *De humani corporis fabrica*, 1555)

The sphenoid bone is an unpaired and symmetric median bone located in the middle of the skull base. It has a complex shape that has been deciphered over several centuries, and for this reason it has received numerous names: the “cuneiform bone” by the Romans (Sawai 2008), because of its insertion as a wedge between the neighboring bones; the “sphenoid” by the Greeks, because it is wedge- or wing-shaped; “os colatorii” by the Arabs, as they believed that the pituitary gland sits on a spongy seat with foramina through which excretions from the brain flow; and the “basilar bone” by the barbarians, as it is located at the skull base (du Laurens 1621). Because of its morphology, the bone has also been called the “sphecoideum” (*Wespenbein*), “vespiforme,” “alatum,” “os carinae,” “polimorphon,” “multiform,” or “pterigoideum” (Spigelius 1627; Hyrtl 1871, 1880). It is a component of the base and the lateral wall of the skull, and the sphenoid connects to all of its bones and to most of the facial bones (Hyrtl 1871). As it is a bone with an “extraordinarily varied form” (Vesalius 1555), with a very irregular and complicated shape (Craigie 1838), we only recall its main elements here: a central portion called the body, with an approximately cubic shape; two triangular edges emerging from the anterior superior part of the body called the lesser wings (ala minor); two half-moon-shaped extensions emerging from the lateral part of its body called the greater wings (ala major); and two processes located vertically on the inferior face of the sphenoid called the pterygoid processes (Fig. 1a, b).

This bony structure was identified early in antiquity, even though only its external appearance was described. Indeed, archeological findings suggest that Aztec priests performed a primitive form of skull base dissection that allowed them to visualize the sphenoid (Lopez-Serna et al. 2012). The ancients compared the sphenoid to a bat, as they considered its middle to be similar to the body and head of a bat, its temporal processes to resemble a bat’s extended wings, and its pterygoid processes to be like a bat’s feet (Craigie 1838; Bell and Bell 1827).

Galen of Pergamon (129–200), considered the father of anatomy, garnered the greatest reputation of any with the four processes corresponding physician in ancient times (Elhadi et al. 2012). He described the anatomy of the sphenoid bone for the first time, comparing it to a wedge, and this similarity was the origin of the term “sphenoid.” In his text *De ossibus* (*About Bones*), when he described the bones of the head, Galen noted that—for the sake of a clear doctrine (*clariotis doctrine*)—one must assume that the upper jaw is different from the sphenoid bone. As the latter has a wedge-like appearance, Galen called it the sphenoid, from *σφήνα*_Greek_ (*sfina*), meaning “wedge,” and *οιδοσ*_Greek_ (*oidos*), meaning “similar to.” According to the translation of Galen’s writings into Latin, the bone was also called the cuneiform bone: *ad cunei* [*cuneus*_Latin_ means “wedge”]* similitudinem structo*, meaning “with a structure similar to a wedge” enclosed by the frontal, temporal, and occipital bones (Galenus 1630).

Despite containing many mistakes, Galen’s writings were the absolute authority in medieval and Renaissance Europe (Sakai 2007), when Andreas Vesalius (1514–1564), based on numerous dissections, described the morphology of the sphenoid bone. Vesalius, who was considered by Riva et al. (2010) to be the “author of the anatomical revolution,” presented almost all of the anatomical knowledge that had been gained up to that point while simultaneously correcting much of it. He highlighted inaccuracies and supplied clear descriptions accompanied by drawings in chapter VI, *De octo capitis ossibus et suturis* (*About the Eight Bones and Sutures of the Head*) (Vesalius 1555), of his wonderful work *De humani corporis fabrica.* His studies during the Renaissance ushered in the “golden century of anatomy,” including that of the sphenoid (Wysocki et al. 2016).

 Vesalius used the term “cuneiform bone” for the sphenoid, and reminded his readers of the Greek name for it: *σφηνοειδής* (*sphenoide*) (Vesalius 1555), which he obtained from Galen’s writings. (Galen’s works became available to Western physicians after the fall of Constantinople, as the scholars of Byzantium migrated to the Italian Peninsula, taking ancient writings with them.)

Considered by Riva et al. as the “author of the anatomical revolution” Riva et al. (2010), Vesalius mentioned that his ancient predecessors had described the sphenoid bone as a “polymorphous” bone that was unpaired and formed part of the skull cavity containing the brain. Upon drawing the contour of the sphenoid based on its sutures with neighboring bones, Vesalius observed that the cuneiform bone (the sphenoid) looked like a flying bird (Fig. 1c) (Vesalius 1555). He also stated that the sphenoid bone was different from the palate bone, and corrected Galen’s view that the sphenoid bone is like a sieve: “ordinary physicians call it *os colatorii* [the colander bone] and likening it to a wedge, they have passed on the tradition that it is *densum ac durum* [dense and hard] but nonetheless have not forgotten the little foramina in it that purge phlegm, they judge the same as they do many things that occur in Galen” (Vesalius 1555).

**Fig. 2:**
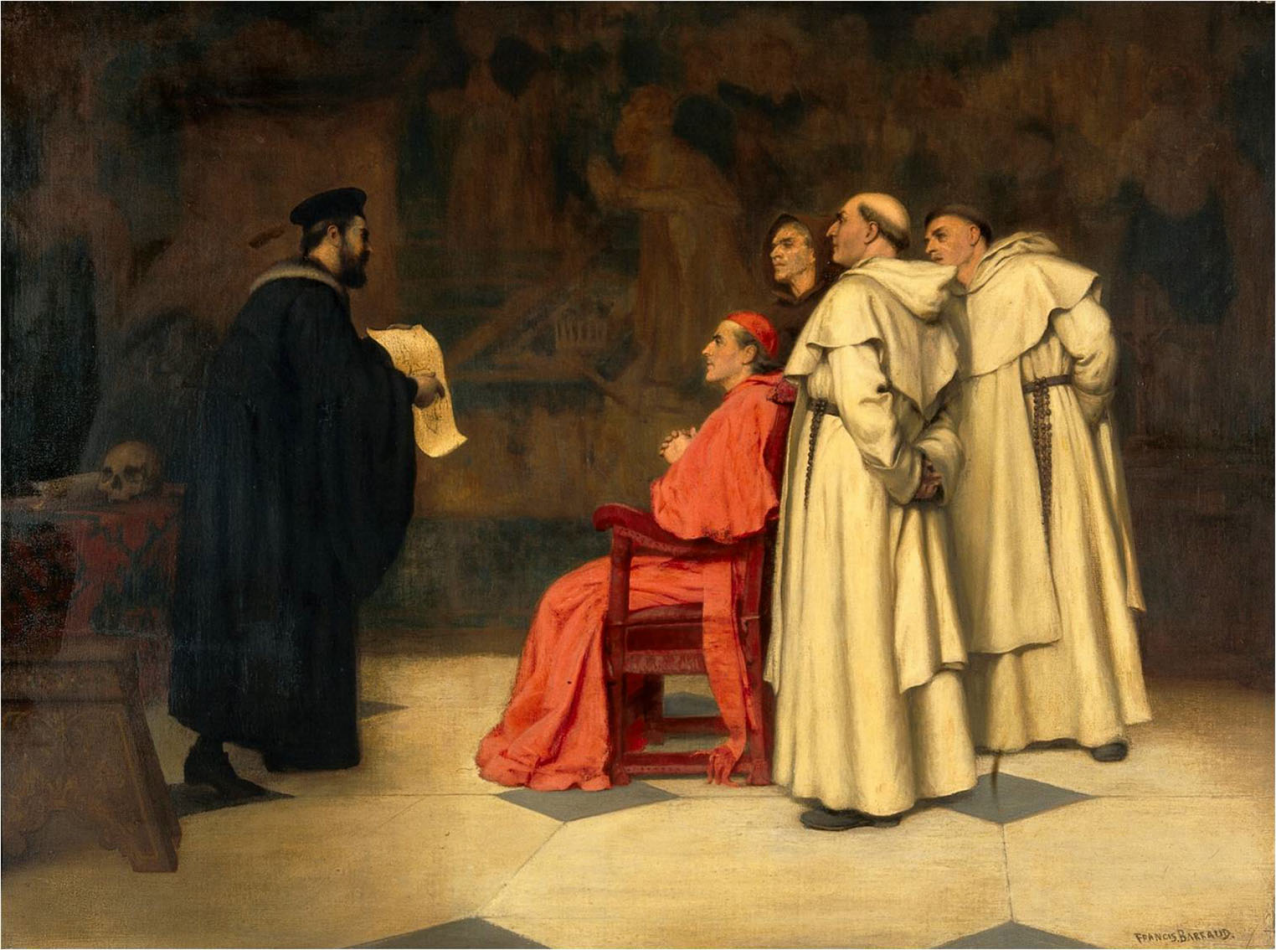
*Gabriel Falloppius, Explaining One of His Discoveries to the Cardinal Duke of Ferrara* by Francis James Barraud (1856–1924), Wellcome Library, London

Another great anatomist who focused on the sphenoid bone was the Italian Gabriele Falloppio (1523–1562), also known by his Latin name Falloppius (Fig. 2), who was a professor of anatomy, surgery, and botany at the University of Padua. He carried out dissections of fetuses, children, and adults. In his work *Observationes anatomicae* (1561), he provided many comments on and corrections of Vesalius’s *De humani corporis fabrica*, as well as other information about the anatomy of the sphenoid bone and its embryology (Fazekas and Kósa 1978).

## The wings of the sphenoid bone

Ancient anatomists initially differentiated only the body and the greater wings of the sphenoid bone (Cloquet and Knox 1828), comparing them with the wings of a bat or bird (Turliuc et al. 2017). Vesalius described the greater wings of the sphenoid bone in detail, but the Sicilian anatomist Giovanni Filippo Ingrassias (1510–1580), Vesalius’s student and later a professor at the University of Naples and Protomedicus of Sicily, gave the first distinct account of the true configuration of the sphenoid bone (Craigie 1838). The illustrious anatomist Arcangelo Spedalieri (1779–1823) stated that Vesalius and Columbo, his student and successor at Padua, sketched the sphenoid bone imperfectly, whereas Ingrasia presented it faithfully and was the first to describe the two lesser wings that were later denoted the processes of Ingrassias in his honor (Spedalieri 1817).

## The sellar region of the sphenoid bone

When Galen studied the sphenoid bone, he identified and described a structure that he called the glandula pituitaria (derived from *pituita*_Latin_, meaning “glairy mucus”) at the level of the upper face of the sphenoid bone body. He stated that this structure was* extra durem matrem posita est* (placed outside the dura mater), and described the depression in the sphenoid bone in which the structure is located (*pituitaria cerebri cava*; Galenus 1630). Based on these findings, Galen formulated the remarkable theory that waste products from the activity of the brain are discharged through this depression in the sphenoid bone (i.e., the sella turcica) and the cribriform plate as phlegm (Greenblatt et al. 1997; Johnson and Green 2014). Galen reached this conclusion because he observed the release of a liquid similar to phlegm from Rathke’s cysts during some dissections of animals (Johnson and Green 2014).

**Fig. 3a–d:**
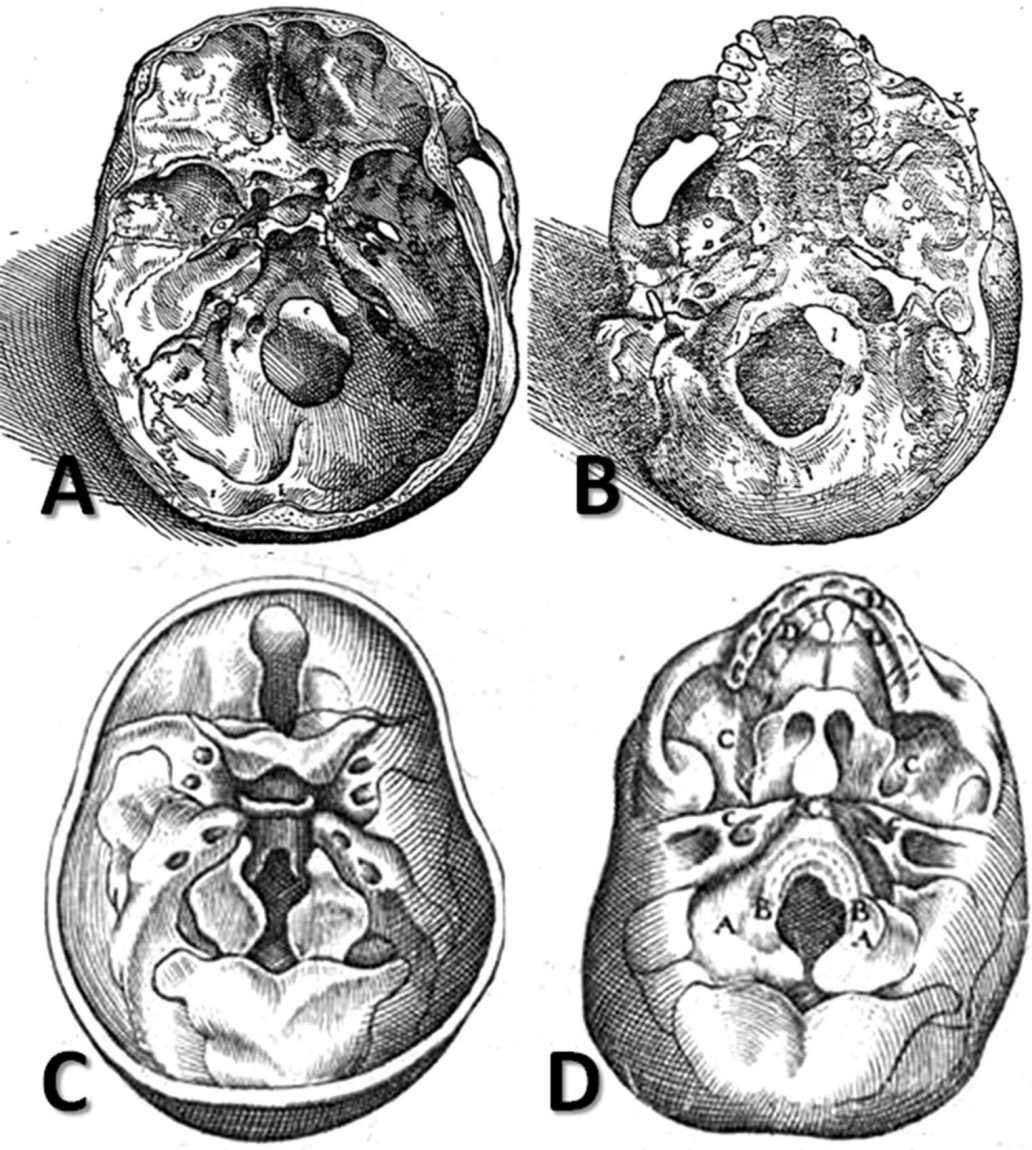
Drawings of the skull base highlighting the sphenoid bone in Vesalius’s *De humani corporis fabrica* (1955) (**a** and **b**) and in du Laurens’ *Historia anatomica humani corporis* (1600) (**c** and **d**)

Fourteen centuries later, in 1543, Vesalius described the anatomy of the entire sellar region (Fig. 3a) and stated that the interior surface of the cuneiform bone had a broad depression containing a gland (the glandula pituitaria) into which phlegm flowed from the brain (Vesalius 1555), and he termed this depression a *sinus.* Moreover, he had the courage to criticize Galen (Toni 2000), asserting that the ancient anatomist’s opinion in this respect was “totally and completely wrong,” as the depression in the sphenoid bone was not perforated “like a sponge or a sieve” as Galen stated but was “solid and continuous” (Vesalius 1555). Vesalius’s repeated attacks on his illustrious predecessor’s opinions led to much criticism of him by Galen’s defenders, to the extent that they nicknamed Vesalius “Vesanus” (madman) (Magner 2002).

In 1559, the Italian anatomist Realdo Columbo (1515–1559) published his masterpiece *De re anatomica libri XV*, dedicated to Pope Paul IV. Columbo asked one of his patients, the brilliant Renaissance painter Michelangelo Buonarroti (1475–1564), to illustrate the book, but this never came to pass due to Michelangelo’s old age at the time it was published (84 years old) (Porzionato et al. 2012; Porter 1985; O’Rourke Boyle 1998). Columbo ignored the term “cuneiform bone” and used *sphenoide* instead. He also noticed a similarity (*sellæ simillimum*) between the depression in the sphenoid and a chair, which led him to introduce the term *sella*_Latin_ for this depression. Just like Vesalius, Columbo corrected Galen’s error regarding the existence of numerous foramina in the sphenoid bone (Columbo 1559).

In 1600, the French anatomist André du Laurens (1558–1609), who was the Rector of the Medical School of Montpellier and *consilarius et medicus ordinarius* (counsellor and physician) to King Henry IV of France and Navarre, published his monumental and erudite work *Historia anatomica humani corporis*. In the second book of that work, *De ossibus*, he allocated two chapters (XIII and XVI) to a description of the sphenoid bone (Fig. 3c, d). He used the term *sella equinæ*_Latin_ for the structure located on the internal face of the sphenoid bone that contained a soft gland because he felt that it looked like the saddle of a horse (*a sellæ equinæ forma*) (du Laurens 1600) (Fig. 3c).

**Fig. 4:**
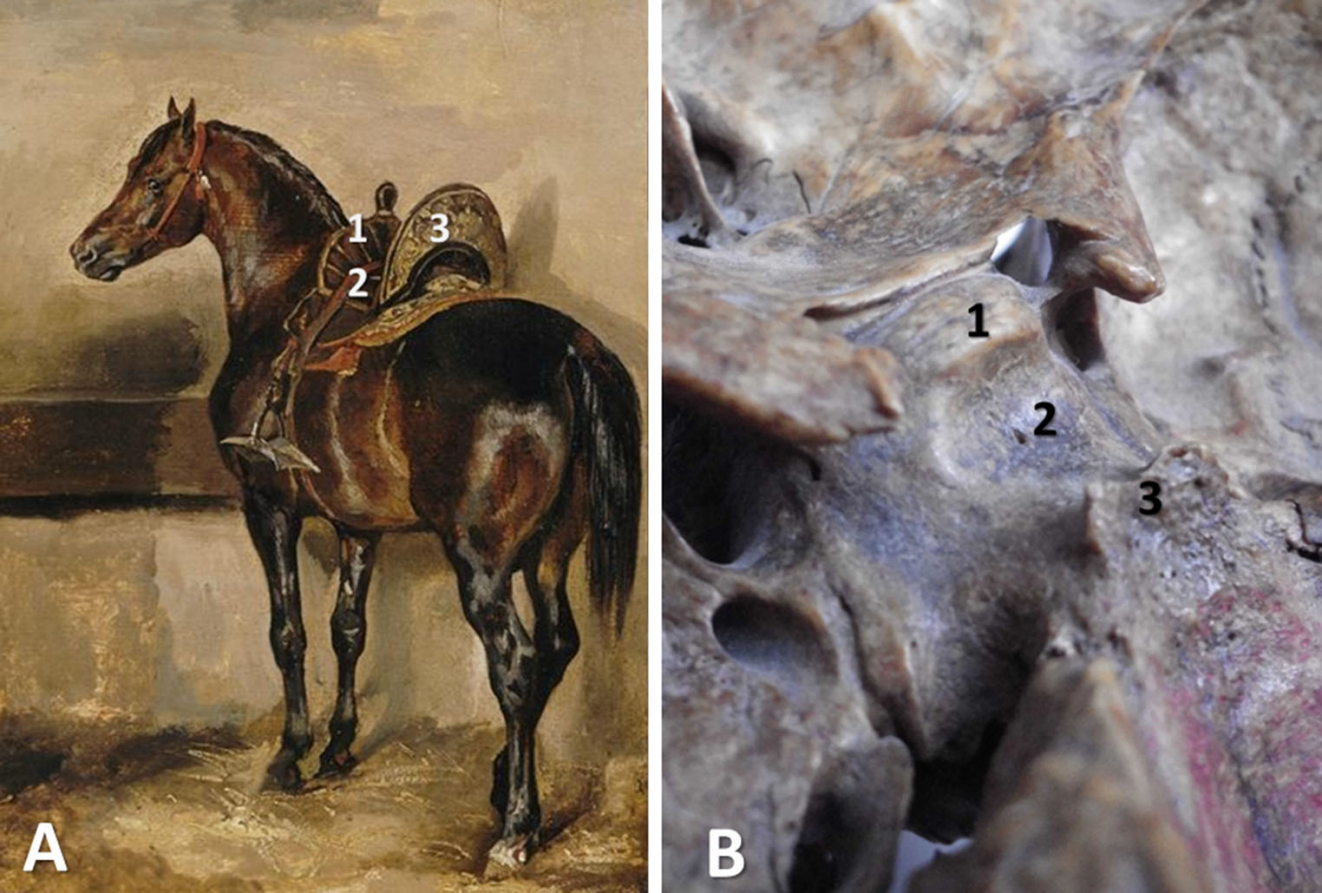
**a** *Turkish Horse in a Stable* by Theodore Gericault (1791–1824); note the labeled parts of the Turkish saddle: pommel (*1*), seat (*2*), and cantle (*3*) (public domain). **b** The anatomy of the sella turcica: tuberculum sellae (*1*), hypophysial fossa (*2*), and dorsum sellae (*3*) (Dr. A. Iordache’s personal collection)

A quarter of a century later, the Flemish physician and botanist Adrianus Spigelius (1578–1625), one of the most eminent anatomists to work at the University of Padua during the seventeenth century, reused the term “cuneiform bone” and described the saddle-shaped depression in its thickest region, comparing it with a Turkish saddle: *extuberantibus, qui cum ossis crassam partem cingant, ephippio non absimilem, Sella turcica a forma dicuntur* (“the protuberances, which are said to be in the shape of a Turkish saddle, because they surround the thick part of the bone, not unlike a saddle”) (Spigelius 1627). The term *sella turcica*_Latin_ (Turkish saddle) was introduced in Spigelius’s famous work *De humani corporis fabrica libri decem* (published in 1627, two years after his death), possibly because the Turkish cavalry was prominent in Westerners’ minds, given that the Ottoman Empire had recently been at the apex of its power under Sultan Suleiman the Magnificent. Indeed, the stength of the Ottoman Empire prompted many curious Europeans to visit Constantinople during this period. One of them was the painter Melchior Lorck (1526/27–1583), a Danish artist who was assigned to the Embassy of the Sublime Porte in 1555 by the German king Ferdinand I (Holy Roman Emperor from 1556). The painter was astonished by the elegance of Turkish saddles and produced many visual records of them. Lorck’s journey resulted in 128 woodcuts, which he intended to publish as a book. However, he did not succeed in this; *The Turkish Publication* (the title of the resulting book) did not come out until 1626, long after Lorck’s death (Lorck 1626; Warner 2012). After the publication of that book, the beauty of Turkish saddles became widely known and influenced artists of the period (Fig. 4a), and even anatomical terminology (Fig. 4b).

A group of Turkish medical historians (Tekiner et al. 2015) recently presented analogies between the Turkish saddle of the seventeeth century and the sella turcica of Spigelius. The sella turcica has three parts. The first is the tuberculum sellae, the slight anterior elevation on the body of the sphenoid bone, which corresponds to the pommel (the upward-curving or upward-projecting part of a saddle in front of the rider). The second part is the hypophyseal fossa, which hosts the hypophysis and resembles the seat of the saddle. The third part is the dorsum sellae, which is similar to the cantle—the raised, curved part at the back of the saddle (Fig. 4a).

In 1998, as a variety of terms were being used in different countries for the saddle-shaped depression on the sphenoid (including “sella turcica,” “sella equina,” “ephippium,” “sella sphenoidis,” and “Turkish saddle”), the Federative Committee on Anatomical Terminology (FCAT) selected “sella turcica” as the official Latin and English term for this anatomical feature to promote international consistency in nomenclature (Tekiner et al. 2015; FCAT 1998).

## Clinoid and pterygoid processes

The pituitary fossa or sella turcica is surrounded at its four corners by four bone prominences (called the clinoid processes or apophyses), two anterior ones (forming parts of the lesser wings), and two posterior ones (where the cerebellar tentorium inserts). The term “clinoid” comes from the Greek words *kλιηι* (*clini*), meaning “bed,” and  (*oidos*), meaning “similar to,” as the ancients thought that the depression on the internal face of the sphenoid bone resembled a tent bed, with the four processes corresponding to the four piles supporting the tent (Cruveilhier 1853; Skinner 1961).

Jacobus Sylvius (1478–1555), Vesalius’s teacher and one of the greatest anatomists of the Renaissance period, was the first to inject the blood vessels in order to examine the pterygoid and clinoid processes of the sphenoid bone (Weinberger 1926). Sylvius recognized only three clinoid apophyses: two anterior and one posterior (Portal 1804).

Continuing with the description of the broad depression of the sphenoid bone containing a gland, Vesalius noted the ancient comparison of the four processes situated around the empty cavity in the sphenoid: “its most prominent parts are four constant processes to which the hard membrane [i.e., dura mater] of the brain is strongly attached and which somehow resemble the lower part of a chariot (*lecticæ mensa*) and are therefore called *κλινοειδεί*_Greek_ (*klinoeidei*, meaning “clinoid processes”)” (Vesalius 1555).

The pterygoid processes were named by Galen in the second century, based on the resemblance of these processes of the sphenoid bone to the wings of a bird (Wain 1958). Andreas Vesalius also described the pterygoid processes of the sphenoid bone (Fig. 3b), comparing them to a bat’s wings: “On its lower surface, where it is rough primarily for the tunic that surrounds the cavity of the nostrils and is attached to the bony nasal septum [i.e., vomer], it puts forth four conspicuous processes, two on each side, thin and prominent like the wings of a bat, called *πτερυγόηδες*_Greek_ [pterygoides] (*vespertilionum allarum modo tenues et proeminentes unde etiam al illarum imagine πτερυγόηδες nuncupantur*)” (Vesalius 1555).

**Fig. 5:**
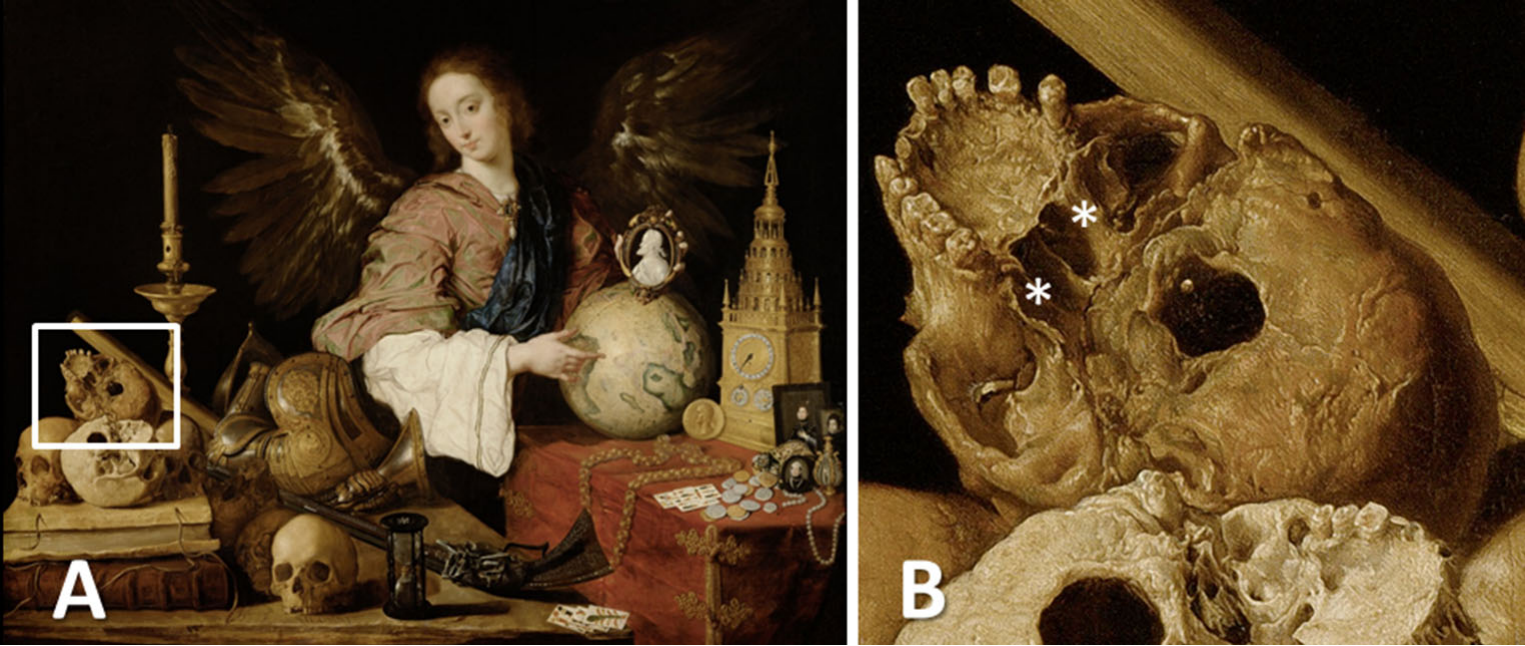
**a** *Vanitas* by Antonio de Pereda y Salgado (1632–1636). **b** Detail showing the pterygoid processes (indicated by *asterisks*) of the skull depicted in the painting (public domain)

The anatomical knowledge of the morphology and structure of the sphenoid bone acquired by anatomists during the Renaissance influenced Baroque painters, who wished to accurately represent the human form (Kemp 2010). Indeed, soon after Vesalius’s remarkable works on the human cranium and consequently the sphenoid bone, sociopolitical events of the period led the artistic community to adopt the skull as a symbol of the impermanence of life (Fig. 5a, b).

## Foramina and bone impressions

In book VII of *De humani corporis fabrica*, Vesalius allocated a whole chapter to the foramina at skull level—*De ossium capitis et maxillae superioris foraminibus. Caput XII* (*Chapter XII. The Foramina in the Bones of the Head and Upper Jaw*)—in order to help students to understand the pathways of the intracranial arteries, veins, and nerves. At the same time, with a certain degree of malice, he referred to the many errors made by other experts in anatomy when they had described these foramina (Vesalius 1555).

Based on a “careful and accurate dissection,” he described the shape of the foramina at the level of the sphenoid bone in detail with the aid of appropriate drawings, labeling all visible anatomical structures at the skull base level with the letters of the alphabet; e.g., “H” for foramen rotundum, “Q” for foramen ovale, and “R” for foramen spinosum (Vesalius 1555) (Fig. 3a, b). Moreover, he included all of the vascular and nervous structures passing through them in his descriptions.

The letter “S” was used by Vesalius to label a distinct but inconstant foramen that was located between the foramen rotundum and the foramen ovale of the sphenoid bone and which was subsequently named after him: the foramen Vesalii (Hoblyn 1865). He discussed this foramen as follows: “Occasionally a small foramen is observed on the inner side of the foramen which transmits the two pairs of nerves just referred to, serving a small branch of the same vein. It appears rarely on one side of the skull and much more rarely on both” (Vesalius 1555).

## The sphenoid sinus

In his treatise *Isagogae breves in anatomiam humani corporis*, the Italian anatomist Berengario da Carpi (1460–1530) also contributed to our understanding of the anatomy of the sphenoid bone, as he established its margins and sutures with neighboring bones (Ball 1910). Moreover, he was the first to report the sphenoid sinuses (Skinner 1961), which later became notorious as the cavities that exhibit the greatest variability of any in the human body (Teatini et al. 1987).

Nevertheless, the clearest information on the sphenoid sinuses was provided by Vesalius, even though he did not believe that they existed in children. In his work *De humani corporis fabrica*, he mentioned that the lower part of the body of the sphenoid had two cavities that he called *antra* and were separated by a bony septum, similar to a wall in the middle of a house (Vesalius 1555). Spigelius also referred—albeit rather vaguely—to these cavities, writing that *sunt etiam sinus plures huic ossi* (“there are also several cavities in the bone”) (Spigelus 1627).

## Conclusions

Due to the polymorphous structure of the sphenoid bone, there have been many anatomists—over the course of millennia—who have made valuable contributions to our knowledge of this structure in the human body. A line in Vesalius’s *De humani corporis fabrica* may explain the continuing fascination of anatomists with the sphenoid: “if you consider such details of the human fabric worth studying and are fascinated by things which, although they have little practical application in the art, yet demonstrate the wondrous ingenuity of the Creator and were undoubtedly studied with zealous care by the ancient professors of anatomy” (Vesalius 1555). Certainly, without the passion and devotion of anatomists, without their desire to know the manner in which this complex machine known as a human being has formed, scientific progress in this field would not have been possible—for instance, increased knowledge of the anatomy of the skull base facilitated the development of skull base surgery (Prestigiacomo and Dagi 2012).
